# The Effect of Professional Oral Care on the Oral Health Status of Critical Trauma Patients Using Ventilators

**DOI:** 10.3390/ijerph19106197

**Published:** 2022-05-19

**Authors:** Ma-I Choi, Sun-Young Han, Hyun-Sun Jeon, Eun-Sil Choi, Seung-Eun Won, Ye-Ji Lee, Chi-Yun Baek, So-Jung Mun

**Affiliations:** 1 Department of Dental Hygiene, College of Software and Digital Healthcare Convergence, Yonsei University, Wonju 26493, Korea; mai.choi@yonsei.ac.kr (M.-I.C.); syhan0724@yonsei.ac.kr (S.-Y.H.); ces2739@yonsei.ac.kr (E.-S.C.); 2 Department of Dental Hygiene, Yeoju Institute of Technology, Yeoju 12652, Korea; yudhjhs@yit.ac.kr; 3 Dental Life Science Research Institute, The Seoul National University Dental Hospital, Seoul 03722, Korea; d5059@snudh.org; 4 Dental Hygiene, NYU College of Dentistry, 345 E. 24th Street, New York, NY 10010, USA; ygee12@yonsei.ac.kr; 5 Department of Nursing, Wonju Severance Christian Hospital, Yonsei University Wonju College of Medicine, Wonju 26426, Korea; er1847@yonsei.ac.kr

**Keywords:** critically ill patients, dental hygiene, dental plaque, inpatient, oral health status, oral hygiene care, ventilator-associated pneumonia

## Abstract

Background: Oral care reduces the incidence of ventilator-associated pneumonia. In addition, it is important that critically ill patients to maintain their oral health in order to restore their quality of life and to receive adequate nutrition after recovery. Objective: The purpose of this study was to evaluate the effect of professional oral hygiene care (POHC) on the oral health status of patients using a ventilator. Methods: Fifty-seven ventilated trauma patients were admitted to a tertiary medical institution. For 5 days, the dental hygienist performed POHC every 24 h along with routine oral hygiene care (ROHC) every 8 h for the experimental group (Exp.) (*n* = 29), whereas only ROHC was provided the control group (Cont.) (*n* = 28). Oral health status was evaluated using a modified bedside oral exam (MBOE). Results: There was no significant difference between the two groups in the total MBOE score up to 48 h after admission. However, the difference between the two groups was significant for MBOE (F = 16.10, *p* = *0*.000), gingiva (F = 6.02, *p* = 0.018), buccal mucosa (F = 4.21, *p* = 0.046), and dental plaque score after 72 h (F = 13.15, *p* = 0.000). Conclusion: This study confirms the importance of POHC in improving the oral health.

## 1. Introduction

Most hospitalized patients have severe underlying conditions and weakened immune systems. Moreover, they are exposed to risks such as various invasive issue and multidrug-tolerant or -resistant sickness pathogens and, therefore, are highly likely to contract secondary infections [1]. In particular, in the case of hospitalized patients in the intensive care unit, many of them are intubated to open their airways, which increases the chances of contracting pneumonia and ventilator-associated pneumonia (VAP) if bacteria colonizing the oropharynx due to poor oral hygiene move to the lungs [2]. Because daily oral care with chlorhexidine has been included in the VAP bundle since 2010, oral care has been consistently provided to patients with severe conditions [3,4], and it has been reported that providing regular oral care regardless of the use of antibiotics not only helps improve oral health conditions but is also reported to be an evidence-based practice that reduces the risk of developing VAP and other complications [5]. Nonetheless, VAP occurrence is still reported [5].

Patients in the ICU are often unconscious and tend to develop dry mouth, especially when they are orally intubated, because they are lying down and their mouths are always open [6]. Furthermore, because various drugs are administered during hospitalization, saliva secretion decreases and water intake is limited depending on the condition of the critically ill, thus leading to dryness of the oral cavity [6]. Dry mouth worsens teeth, tongue, and oral cavity problems and promotes bacterial activities within the mouth, which results in dental decays, periodontal disease, and halitosis [7]. Additionally, dry mouth is a risk factor for mastication difficulty and swallowing disability, which adversely affect the intake of proper nutrition in the future [8]. Therefore, it is crucial to perform oral care for maintaining good oral hygiene.

It is very important to maintain oral health via adequate oral care, chewing food well, and ingesting it to obtain sufficient nutrients, which forms the basis for health management. The poor oral condition of patients is closely related to poor nutritional status, and it also affects their physical and psychological well-being and quality of life. Poor oral health may also have an impact on the recovery of a patient as well as on the length of hospitalization [9]. However, it is difficult for most patients in the ICU to independently perform activities, such as tooth brushing, for maintaining oral hygiene. Therefore, the provision of professional oral care during a period when it is difficult for patients to perform self-oral care is extremely important for the maintenance of good oral health [5,10]. In this study, it is important to provide oral care to inpatients in order to prevent oral diseases, and to provide appropriate oral care, we reported the need to include dental professionals in the hospital ward staff [11].

Patients with trauma are especially vulnerable to infections, and more efforts should be taken to prevent the occurrence of nosocomial infections [12]. Currently, many studies are being conducted on the importance of oral care in patients with stroke, the elderly, and patients hospitalized for cancer treatment [5]; however, studies on the oral hygiene care of patients with severe trauma are insufficient. In addition, no research has been conducted on the effect of professional oral hygiene care on the health status of patients with severe trauma, which has resulted in insufficient evidence for the development of a systematic oral care protocol.

Thus, this study intends to evaluate the effect of professional oral hygiene care on the oral health condition of patients with trauma who are on ventilator support and are at a high risk of worsening oral health.

## 2. Materials and Methods

### 2.1. Study Design

This study is an interventional study and a non-randomized comparative clinical trial study. This study was reviewed and approved by the institutional review board of Yonsei University Committee of Wonju Severance Christian Hospital on 22 November 2017 (approval number: CR317109). This trial was registered in the Clinical Research Information Service [Internet]; Osong (Chungcheongbuk-do): (KCT0005275), which is affiliated with the WHO International Clinical Trial Registry Platform.

### 2.2. Study Participants

**Figure 1:**
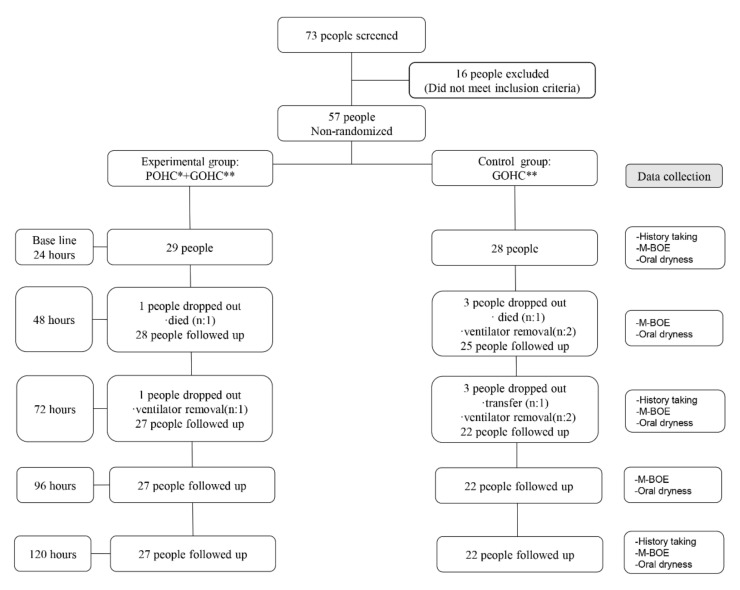
Study process.* POHC = Professional oral hygiene care by dental hygienist; ** GOHC = General oral hygiene care by nurse.

Among the trauma patients admitted in the intensive care unit, patients on ventilator support in tertiary medical centers located in Wonju, Kangwon from 28 February to 1 November 2018 were included in the study. Using the G*Power 3.1 program, the F test was calculated according to the measurement index. Sample sizes were determined for the probability of a Type I error α = 0.05, power 1 − β = 0.95. Effect size f was 0.35, number of groups = 2, and corr among rep measures = 0.5. The total sample size was 70. In consideration of the dropout rate of the experimental study, a total of 100 people were counted, and the experimental group and control group were each 50 people. Patients were cross-assigned into the experimental group and control group based on their hospitalization order. The inclusion criteria for the patients were as follows: those who were admitted to the intensive care unit due to trauma, aged ≥20 years, on ventilator support, who were judged as requiring oral care by the doctor in charge. Those who or whose legal guardian did not agree to participate in the study, those who could not participate due to the severity of their condition, and those who could not receive oral care due to severe injury to their mandible were excluded (Figure 1).

### 2.3. Examination of Oral Health Condition and Performance of Professional Oral Hygiene Care

#### 2.3.1. Examination of Oral Health Condition

In this study, a total of three dental hygienists alternately inspected the oral health status of the experimental group and the control group, and the same examiner examined the oral health status of patients from rounds 1 to 5 if possible. The three examiners looked at various photos of oral conditions found through Google image search, evaluated them with the MBOE index used in this study, and compared them with each other. The training activity was performed thrice or until the same results were obtained. Finally, 35 pictures were selected, and intraclass correlation coefficients (ICCs) were obtained; this value was 0.899.

#### 2.3.2. Performance of Professional Oral Hygiene Care

A professional oral hygiene care protocol was prepared by the researcher by referring to the existing literature and ongoing studies. The prepared protocol was sent to other occupational groups (the nurse in charge and doctors of the ICU for patients with trauma, dentists and dental hygienists of the oral and maxillofacial surgery department, and dental hygienists in charge of managing the oral hygiene of patients admitted in Ichikawa hospital) for review and revision. Based on the revised protocol, a professional oral hygiene manual was prepared by modifying the items and detailed protocols used after discussion among the researchers. For professional oral hygiene care, a dental hygienist provided oral care using a pediatric toothbrush (small head, fine hair/e-Clean mom & kids #515 (EoS)), CHX 0.12% solution and cotton balls and six dental hygienists performed it in turn. Regarding the toothbrushing method, CHX 0.12% solution was applied to the toothbrush, the bristles were placed on the gingival margin, and light vibrations were applied to brush. If there were wide gingival embrasures, the dental plaque between teeth was removed with an interdental brush. The buccal mucosa, tongue, and palate were wiped with a cotton ball soaked in CHX, and hardened sputum adhering to the mucous membrane was dissolved and removed. Suction was continued to prevent the solution from flowing into the airways.

When cleaning the buccal mucosa, it stimulates the parotid and submandibular glands to induce salivation. For patients with dry lips, Vaseline was applied to the lips, and if the oral cavity was dry, Dry Mund (Dong-A pharm., Korea) was applied using a cotton ball to help moisturize. Before the start of the study, after practicing on a low-fidelity simulation model, a total of 3 repeated simulations were performed using the manual for 4 healthy adults without systemic diseases.

#### 2.3.3. Training for General Oral Hygiene Care

The purpose and method of the study were explained to the nurses who performed general oral hygiene for patients with direct trauma in the intensive care unit, and protocols for general oral hygiene care were provided so that oral care could be provided to patients in the same way. General oral care was performed by a nurse every 8 h. It is a method of cleaning the teeth and oral mucosa by moistening a disposable oral swab (Hummed 0-4043-02 Sponge/molten) with 0.12% chlorhexidine. Training was given using printed materials and face-to-face education in groups. Furthermore, additional videos on the oral care method were distributed so that the nurses could refer to them any time.

#### 2.3.4. Intervention Performed for Each Group

In the experimental group, starting from 24 h after ventilator support was provided, nurses provided general oral care at an interval of 8 h and dental hygienists performed professional oral hygiene care at an interval of 24 h in accordance with the prepared protocol. In the control group, nurses provided general oral care at an interval of 8 h starting from 24 h after ventilator support was provided. The intervention was performed for 5 days for both the groups (Figure 1).

#### 2.3.5. Study Variables

General patient characteristics and medical history.

General patient characteristics included gender and age, and for medical history, the use of antibiotics was investigated through medical records.

#### 2.3.6. Modified Bedside Oral Exam (MBOE)

In order to check the oral health condition of the patients, the Bedside Oral Exam (BOE) [13], which Prendergast et al. used to evaluate patients’ oral condition, and the Oral Exam Guide (OEG) [14] developed by Beck S. were modified and used. Among the eight categories in the BOE, this study used the lips, saliva, teeth and denture, and halitosis categories as they were. The degree of plaque deposition was judged as 0 points for clean teeth, 1 point for localized plaque between teeth, 2 points for general interdental area or localized plaque deposits, and 3 points for extensive tooth and interdental plaques. The gingiva, buccal mucosa, and tongue, which are categorized as soft tissue, were classified into texture, color, and moisture in the OEG and used by appropriately combining two indicators for patients with endotracheal intubation admitted in the ICU. The total score ranged from 0 points (very good oral condition) to 42 points (very poor oral condition), and for each item, the average score ranged from 0 points to a maximum of 3 points.

### 2.4. Analysis Method

General characteristics and medical history of the patients were analyzed using frequency analysis and independent *t*-test. To examine the oral health condition over time, repeated measures analysis of variance was performed. Statistical analysis was performed using the SPSS 21.0 program, and the significance level was α = 0.05. For group comparisons by period, post hoc analysis was performed using the independent *t*-test, and the statistical significance level was corrected to 0.01 using Bonferroni correction to reduce the type I error. This study reanalyzed oral health condition-related variables from the “Influence of professional oral hygiene care on reducing VAP in trauma ICU patients”, which is supported by the National Research Foundation of Korea.

## 3. Results

### 3.1. General Patient Characteristics and Oral Health Status

The average age of the patients in the experimental group was 60.62 ± 15.67 years and that of those in the control group was 57.43 ± 16.94 years. There was no significant difference among the patients in terms of age, sex, or use of antibiotics. Additionally, there was no significant difference between the experimental and control groups in terms of the lips, gingiva, tongue, and the presence of plaque and odor, which verified the homogeneity of the sample (Table 1).

### 3.2. Changes in the Oral Health Status of the Patients According to Professional Oral Hygiene Care

**Figure 2:**
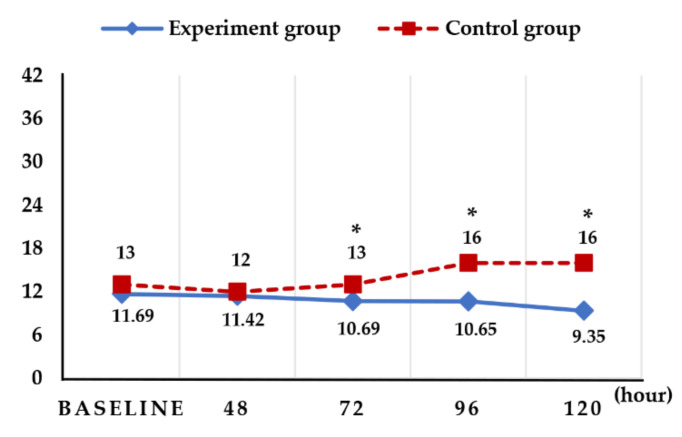
Changes in the M-BOE score. As a result of analyzing the difference in BOE score between rounds, there was no significant difference between the experimental group and the control group at 24 and 48 h, but there was a significant difference between the two groups at 72, 96, and 120 h (an independent *t*-test was conducted at each time point, Bonferroni correction * *p* ≤ 0.01).

Following the calculation of the BOE scores at 24 h (T1), 48 h (T2), 72 h (T3), 96 h (T4), and 120 h (T5) after hospitalization, no significant differences were found between the BOE scores obtained at each evaluation point in the experimental group (scores at T1, T2, T3, T4, and T5 were 11.69, 11.42, 10.69, 10.65, and 9.35, respectively (F = 1.41, *p* = 0.242)). However, there was a significant difference in the BOE scores depending on the oral health care status (F = 16.10, *p* = 0.000). Additional analysis of the difference between BOE scores obtained at each evaluation point revealed no significant difference between experimental and control groups at T1 (within 24 h) and T2 (after 24 h); however, a significant difference was found between the two groups at T3, T4, and T5 (Figure 2).

### 3.3. Changes in the Oral Health Status by Items According to Professional Oral Hygiene Care

Changes in soft tissue scores.

**Figure 3:**
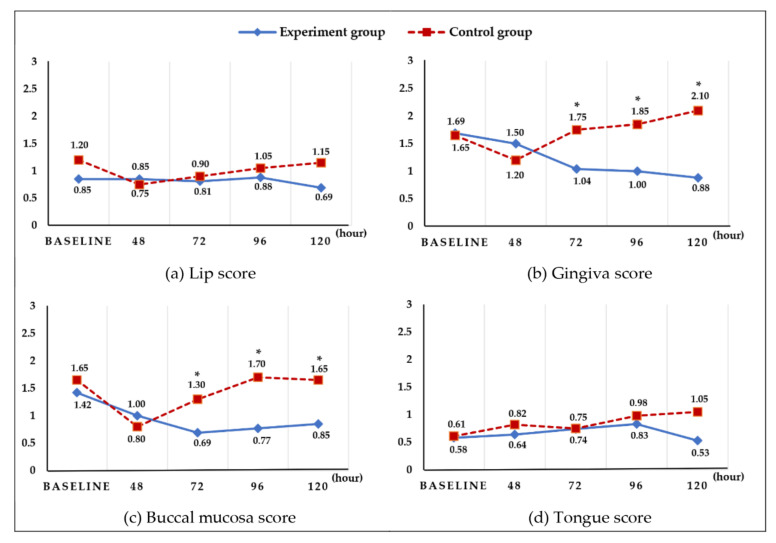
Changes in the oral area (soft tissue) score. There were no significant differences in lips and tongue, but significant differences were observed between groups in gingiva and buccal mucosa at 72, 96, and 120 h (an independent *t*-test was conducted at each time point, Bonferroni correction * *p* < 0.01).

Examination of the soft tissues at 24 h (T1), 48 h (T2), 72 h (T3), 96 h (T4), and 120 h (T5) after hospitalization revealed no significant difference in the lips and tongue, whereas a significant difference was found between the two groups in terms of the gingiva (F = 6.02, *p* = 0.018) and buccal mucosa (F = 4.21, *p* = 0.046) at 72 h (T3), 96 h T4) and 120 h (T5) (Figure 3).

Change in saliva, presence of plaque, and odor scores.

**Figure 4:**
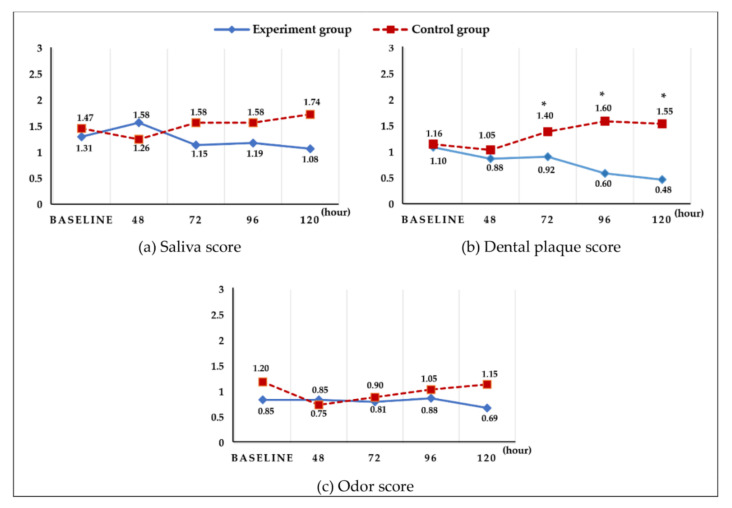
Changes in saliva, dental plaque, and odor score. There was no significant difference in saliva and odor, but the degree of dental plaque showed significant differences between groups at 72, 96, and 120 h hours (an independent *t*-test was conducted at each time point, Bonferroni correction * *p* < 0.01).

Examination of the saliva, presence of plaque, and odor at 24 h (T1), 48 h (T2), 72 h (T3), 96 h (T4), and 120 h (T5) after hospitalization showed no significant difference in the saliva and odor scores, whereas a significant difference was found between the two groups in terms of the presence of plaque at 72 h (T3), 96 h (T4), and 120 h (T5) (F = 13.15, *p* = 0.000) (Figure 4).

## 4. Discussion

This study evaluated the results of the two groups of patients with trauma admitted to the ICU—one that received professional oral care for 5 days and the other that did not receive it. It was confirmed that the oral condition of the patients in the experimental group who received professional oral hygiene care was improved compared to the control group.

Systematic literature review and meta-analysis results on the oral care of patients on ventilators [5] showed that there were differences in the oral care protocols and tools being used in ICUs depending on the hospitals and oral care providers. Additionally, even though oral conditions should be assessed before providing oral care, most of the existing tools have been developed for elderly patients or for those with cancer, thus making them unsuitable for patients in the ICU who must be assessed quickly and accurately. Therefore, in this study, the BOE and OEG were modified and used to evaluate the oral health status of patients. Oral health status evaluation at hospitalization confirmed that there was no difference between the experimental and control groups. However, there were deviations in the overall MBOE, gingiva (corresponding to soft tissue), and mucosal condition scores of the patients. Because patients’ oral health conditions vary, it is necessary to select those with poor oral hygiene with appropriate assessment tools at the beginning, perform careful observation, and provide appropriate oral care, especially for the soft tissues. Moreover, since the oral health status evaluation tool used in this study was developed for inpatients, it is necessary to develop appropriate tools in the future for patients on ventilator support to overcome the difficulty in evaluating “dysphagia” and “saliva” in them.

Examining the changes in oral health status revealed that the experimental group that received professional oral hygiene care by dental hygienists at 24 h intervals showed a significantly improved oral health status compared with the control group. After 48 h, the experimental group significantly differed from the control group, especially in terms of soft tissues, such as the gums and mucosa, and plaque deposition, thereby indicating that professional oral hygiene care using toothbrush and chlorohexidine was effective.

The most efficient way to remove dental plaque, which is the cause of dental decay and periodontal disease, is the use of a toothbrush [15,16]. In this study, first, a chlorohexidine solution was coated on a toothbrush meant for pediatric use. Next, the toothbrush was placed at the gingival margin, and gentle but light vibratory strokes were used. In a study by Nakahodo et al. [17], among patients admitted to the internal medicine ward, the number of bacteria decreased significantly in the group using a toothbrush to wipe the mucous membrane compared with the group using a gauze or sponge. Gingivitis has been shown to be associated with dental plaque deposition around the gingiva, and bacteria live in various areas in the oral cavity. Particularly, the gingivitis-causing bacteria living in the gingival cells are an important cause of periodontal disease [18]. In this study, it is thought that the overall oral health condition was improved by effectively removing the dental flora along the interdental space and gingiva using a toothbrush and by massaging the mucous membrane and gums using a cotton ball.

In this study, the oral health condition was improved after providing regular oral care by nurses every 8 h and performing professional oral hygiene care by dental hygienists every 24 h. The results obtained after 72 h showed differences for each group. Terezakis et al. [19] reported in their study that the oral health condition is aggravated as the amount of dental plaque within the mouth increases during 7–20 days after hospitalization. Furthermore, bacterial colonies in the oropharynx have been reported to be one of the main risk factors for VAP. Efforts are being made to remove and prevent bacterial deposition in the oral cavity via oral care in the ICU [4,5]. Bacteria residing in the mouth or pharynx of the general public do not cause pneumonia. However, in critically ill patients, it has been reported that the main bacterial population changes from Gram-positive to VAP-causing bacteria and that more harmful Gram-negative bacteria emerge [20]. When a ventilator is intubated, the patients’ microflora moves rapidly to the lower respiratory tract, which increases the risk of pneumonia development [21]. Therefore, timely professional oral hygiene care is necessary in addition to regular oral care for maintaining a good oral health condition and preventing systemic problems. Evidence-based evaluation tools should be developed to assess the oral health status of patients in the ICU and to determine the appropriate time for performing professional oral hygiene care.

It has been reported that providing oral care to hospitalized patients can prevent respiratory diseases, such as pneumonia, and that it can help maintain good oral condition, swallowing condition, and nutritional status, thus resulting in a higher discharge rate [22,23]. Patients in the ICU often require continuous observation because of the severity of their disease and the high mortality rate. They are transferred to the general ward according to the judgment of medical staff when their physical condition is stabilized and when they do not require intensive care or mechanical support [24]. As oral ingestion becomes possible in the general ward, the patient gradually recovers. Therefore, maintaining oral health in the ICU not only reduces the risk of infection but also maintains chewing function to quick return to normal conditions after recovery and to promote faster recovery. 

Currently, studies continue to report that multidisciplinary action is required when providing oral care to hospitalized patients and that people with expertise in the dental field should be included [25]. Of late, domestic and international medical communities have been providing the best medical service to patients via the use of a multidisciplinary approach in which experts from various fields gather to collectively determine patient treatment plans. This approach has shown a positive effect on treatment results [26]. In Japan, since 2006, inpatients, patients with cancer undergoing surgery, and patients on ventilator support have been receiving oral hygiene care before and after surgery [27]. Various occupational groups, such as nurses, rehabilitation therapists, dental hygienists, and pharmacists, have cooperated to increase their expertise in each area, and dental hygienists provide professional oral hygiene care. Therefore, a multidisciplinary approach must be developed in the future, which includes dental care professionals for providing oral care to hospitalized patients and those with severe conditions.

The limitation of this study was that a small number of participants were included because some patients did not meet the selection criteria due to different conditions, rapid changes in health status, and various variables (death, extubation, and transfer), making it difficult to conduct the study. The study had a high dropout rate (about 34%; 25 out of 73 patients were excluded); therefore, it is difficult to generalize the results. In addition, since the subjects of this study were equipped with ventilators, it was difficult to observe the oral cavity, and there was a limitation in accurately evaluating oral health status. Therefore, an appropriate oral health status evaluation index that can be applied to a patient who is equipped with a ventilator in the future should be developed. Nevertheless, this study included hospitalized patients with trauma on ventilator support in the ICU and confirmed that their oral health conditions improved as a result of providing oral care in collaboration with various healthcare professionals. In order to develop specific guidelines such as protocols for professional oral hygiene management in the future, a long-term study with a sufficient number of study subjects is required. In addition, in order to reduce the side effects of antiseptic agents, research should be conducted considering various agents such as paraprobiotic and postbiotic gel.

## 5. Conclusions

It was confirmed that oral health condition improved by providing professional oral hygiene care to patients equipped with a ventilator. In particular, it was effective for soft tissue care and dental plaque removal.

## Figures and Tables

**Table 1:** General patient characteristics and oral health status.

Characteristics	Categories	Exp. Group (*n*:29)		Cont. Group (*n*:28)		x^2 †^ or *t* ^‡^	*p*
		*n* (%)	Mean ± SD	*n* (%)	Mean ± SD		
Age (yr)			60.62 ± 15.67		57.43 ± 16.94		0.464
Sex	Male	20 (69)		19 (67.9)		0.008	0.928
	Female	9 (31)		9 (32.1)			
Antibiotics	Yes	25 (86.2)		27 (96.4)		1.860	0.352
	No	4 (13.8)		1 (3.6)			
Oral status							
Lips			0.85 ± 0.78		1.20 ± 0.95	0.161	0.767
Gingiva			1.69 ± 1.44		1.65 ± 1.23	0.281	0.598
Buccal mucosa			1.42 ± 1.50		1.65 ± 1.90	1.411	0.240
Tongue			1.73 ± 1.37		1.84 ± 1.36	1.844	0.176
Saliva			1.31 ± 0.79		1.47 ± 0.96	2.959	0.091
Teeth (plaque)			1.16 ± 0.69		1.10 ± 0.79	0.017	0.895
Odor			0.65 ± 0.80		0.80 ± 0.83	0.942	0.336

Exp. = Experimental group; Cont. = Control group; ^†^ Chi-squared test, ^‡^ independent *t*-test, *p* < 0.05.
